# Does the limb lengthening reduce the incidence of hip dislocation in patients with neurological disorders and insufficient muscle tension who undergoing hip arthroplasty?

**DOI:** 10.3389/fsurg.2024.1259039

**Published:** 2024-05-30

**Authors:** ZiHang Li, Kun Chu, Meng Yang, SiKai Liu, Bo Liu, HuiJie Li

**Affiliations:** Department of Osteonecrosis and Hip Surgery, the Third Hospital of Hebei Medical University, Shijiazhuang, Hebei, China

**Keywords:** total hip arthroplasty, dislocation, muscle tension, complication, limb-length discrepancy

## Abstract

**Background:**

The soft-tissue tension is closely associated with postoperative hip dislocation in patients undergoing total hip arthroplasty (THA), especially for those patients with neurological disorders and insufficient muscle tension. The aim of this study is to explore the effect of limb lengthening on the incidence of complications following THA in patients with neurological disorders and insufficient muscle tension.

**Methods:**

This retrospective analysis examines individuals with neurological disorders, such as ischemic stroke and poliomyelitis, who underwent primary total hip arthroplasty (THA) at our medical center between January 2015 and April 2021. Demographic and baseline characteristics (such as age, gender, muscle strength) were obtained from medical records. The limb length, offset and the positional parameters of both acetabular and femoral component were measured on pre- and postoperative plain radiograph. The primary outcome was the occurrence of hip dislocation. The secondary outcome included the incidence of other complications and the hip function (determined by Harris score). The correlation between the occurrence of hip dislocation and limb lengthening was analyzed.

**Results:**

A total of 258 patients were finally analyzed. The hip dislocations were identified in 35 patients (overall incidence = 13.57%). The incidence of early dislocation was lower in patients whose limb-length discrepancy (LLD) was over 20 mm (incidence = 4.1% for LLD >20 mm, 12.2% for LLD 10 mm–20 mm and 17% for LLD <10 mm). The odds ratio (OR) was 0.206 and 95% confidence interval (CI) was 0.058–0.737 (compared between LLD <10 mm and LLD >20 mm). But the no difference was identified regarding on the incidence of late dislocation among patients with different LLD. Moreover, the overall incidence of other complications was elevated in patients with LLD >20 mm (incidence = 17.58% for LLD >20 mm, 11.11% for LLD 10 mm–20 mm and 3.19% for LLD <10 mm; OR = 6.464, 95% CI = 1.768–23.640). And the Harris scores, which reflected the hip function, was gradually decreased with the increasing in LLD. In terms of the relationship between the offset and dislocation rate, it was found that increased offset discrepancy was associated with decreased dislocation incidence (incidence = 4.71% for offset discrepancy >10 mm, 12.5% for offset discrepancy 5 mm–10 mm and 17.20% for offset discrepancy <5 mm; OR = 0.238, 95% CI = 0.076–0.742). Furthermore, increased offset discrepancy also bring a reduction in late dislocation. The incidences of late dislocation were 0%, 2.5% and 10.8% for offset discrepancy >10 mm, offset discrepancy 5 mm–10 mm and 17.20% for offset discrepancy respectively. Different from that of LDD, the incidences of other complications were similar among patients with different offset discrepancy. Besides, no influence of offset discrepancy on the hip function was identified in this study.

**Conclusion:**

Unfortunately, although increasing in limb length could partially reduce early dislocation postoperatively, it could not affect the incidence of late dislocation in those patients with neurological disorders and insufficient muscle tension. Moreover, over limb lengthening was associated with other postoperative complications and worse hip function. Instead, additional offset could reduce the probability of postoperative dislocation, without increasing the incidence of other complications. Therefore, femoral stem with lower cervico-diaphyseal angle (higher offset) should be recommended to patients with neurological disorders who were in high risk of postoperative dislocation. Isolated increasing in limb length should be avoided.

## Introduction

Total hip arthroplasty (THA) is a crucial modality for treating hip joint diseases that result in functional impairment, effectively addressing hip pain and restoring joint function. However, complications such as infection, prosthesis loosening, fractures, leg length discrepancy, and dislocation are commonly associated with this surgical procedure (1, 2). Among these, postoperative prosthesis dislocation is the second most prevalent complication following THA (3), with soft tissue tension being one of the crucial factors influencing its occurrence. Research has indicated that the soft tissue tension in patients experiencing recurrent dislocations following total hip arthroplasty (THA) is roughly a quarter of that observed in patients who do not encounter dislocations (4). In patients presenting with coexisting neuromuscular afflictions impacting the hip (such as cerebrovascular accident, polio, Parkinson's, cerebral palsy), the propensity for dislocation may be elevated due to muscular laxity or imbalances in hip musculature (5). Studies have indicated that the dislocation rate after initial hip replacement surgery in general patients ranges from 1% to 7% (6), while neuromuscular disorder patients have a dislocation rate as high as 13% within the first three months postoperatively (7).

Prolonging limb length can introduce additional soft tissue tension (1), but whether it can enhance hip joint stability and reduce dislocation risk among patients with neuromuscular disorders remains uncertain. Furthermore, excessive limb lengthening may lead to a range of symptoms such as back pain, spinal curvature, pelvic tilt, and knee joint osteoarthritis (8). Thus, the primary aim of this research is to examine if extending limb length can mitigate the risk of dislocation and its associated complications in patients with neuromuscular disorders. Additionally, a secondary objective is to explore the potential impact of other variables on reducing the likelihood of dislocation. Our hypothesis posits that lengthening the limbs can effectively lower the risk of dislocation following total hip arthroplasty (THA) surgery in individuals with neuromuscular disorders.

## Methods

In general, this study is a retrospective analysis conducted on patients with neuromuscular diseases who underwent THA surgery at the Third Hospital of Hebei Medical University from January 2015 to April 2021. The patients were divided into dislocation and non-dislocation groups based on whether they experienced dislocation after surgery.

Inclusion criteria:

(1) Patients with end-stage hip joint diseases requiring total hip arthroplasty (THA) for treatment, such as: unilateral femoral head necrosis with Association Research Circulation Osseous (ARCO) stage 3–4 or, osteoarthritis with Kellgren–Lawrence (KL) grade 3–4, developmental dysplasia of the hip (DDH) with associated traumatic osteoarthritis, subcapital femoral neck fracture.
(2) Patients with insufficient hip abductor strength due to neuromuscular disorders such as cerebrovascular disease, poliomyelitis, Parkinson's syndrome, and cerebral palsy prior to hip replacement surgery.
(3) Patients undergoing unilateral total hip arthroplasty for the first time.
(4) Patients with a follow-up duration of 2 years or more.
(5) Patients aged 30–80 years

Exclusion criteria:

(1) Patients with hip disease and neuromuscular disease affect different sides of the hip joint.
(2) Patients who have previously undergone other hip surgeries.
(3) Patients with incomplete medical records or radiographic images.
(4) Patients with lower limb shortening or imaging measurements of LLD <0.

Observation indicators: Whether postoperative hip dislocation occurs, as well as the timing of dislocation (within 4 weeks is considered early dislocation, while beyond 4 weeks is classified as late dislocation), and other potential complications due to limb lengthening after THA (Such as back pain, scoliosis, pelvic tilt, knee osteoarthritis), can be definitively ascertained during follow-up.

This study conducted a retrospective analysis using anonymous data, which has been approved by our hospital's ethics committee, exempting the requirement for informed consent.

### Surgical technique

THA surgery was performed by experienced orthopedic surgeons who had received specialized training. General anesthesia or combined spinal-epidural anesthesia was used during the surgery. The patient was positioned in the lateral decubitus position, with the pelvic locator placed between the posterior (sacrum) and anterior (pubic symphysis). The surgery utilized a posterior-lateral approach, with the short external rotator muscles and joint capsule being cut. The hip joint was then dislocated posteriorly to fully expose the acetabulum and proximal femur. Acetabular preparation and implantation were performed first, followed by femoral preparation and implantation of a longer femoral component. Finally, the wound was cleansed, ensuring the correct placement of surgical dressings, and following the meticulous suturing of the external rotator muscle group and joint capsule, the wound will be meticulously closed layer by layer. The prostheses utilized in the procedure were exclusively artificial total hip joint prostheses, all of which were non-restrictive. The majority boasted ceramic-ceramic contact surfaces, while a smaller fraction featured metal-polyethylene contact interfaces.

### Postoperative management and rehabilitation

Prophylactic cephalosporin antibiotics were administered preoperatively and continued for 24 h postoperatively. The affected limb was maintained in an abducted neutral position, and a rotational prevention shoe was used for fixation. Low molecular weight heparin sodium was injected for anticoagulation. After discharge, oral anticoagulants were used for 35 days to prevent thrombosis in the affected limb. If the patient is ambulatory preoperatively or pre-injury and is well-nourished postoperatively, it may be appropriate in the early postoperative period, under the guidance of a rehabilitation therapist, to use crutches to stand and gradually commence ambulation. Discharged on the fifth day after surgery. All patients were advised to refrain from engaging in activities that could lead to the hip joint being positioned in flexion exceeding 90 degrees, or adduction, or rotation beyond the midline. Outpatient follow-up visits are recommended at 1, 3, 6, and 12 months postoperatively, followed by annual follow-ups.

### Baseline data collection

Patient's demographic information including gender, age, hip joint disease, neuromuscular disease, preoperative hip abductor muscle strength, were recorded. The preoperative strength of the abductor muscles was evaluated using the Codex muscle strength rating standard. The patient was assessed by a primary attending physician upon admission and subsequently by a chief physician, while in a supine position during the measurement. The diameter of the femoral head of the prosthesis used was recorded, and The Harris Hip Score (HHS) at 1 year postoperatively was recorded to evaluate the functional recovery of the hip joint, considering pain, function, deformity, and range of motion.

### Radiographic evaluation

The patient underwent anteroposterior and lateral radiographs of the pelvis both preoperatively and on the third postoperative day, and the first postoperative follow-up was performed with CT of both hips. Measurements were performed using standardized anatomical landmarks. Two experienced orthopedic surgeons independently obtained the data using our hospital's image archiving and communication system, and the data were averaged for analysis. For data with large differences, repeat the side measurement and take the average of this measurement. Measurements included Acetabular shell inclination, Acetabular shell anteversion, Femoral stem alignment, Femoral stem anteversion, Limb-length discrepancy, Offset discrepancy. LLD in this study was due to THA, so all measurements of LLD were done in anteroposterior pelvic x-ray is sufficient.

**Figure 1:**
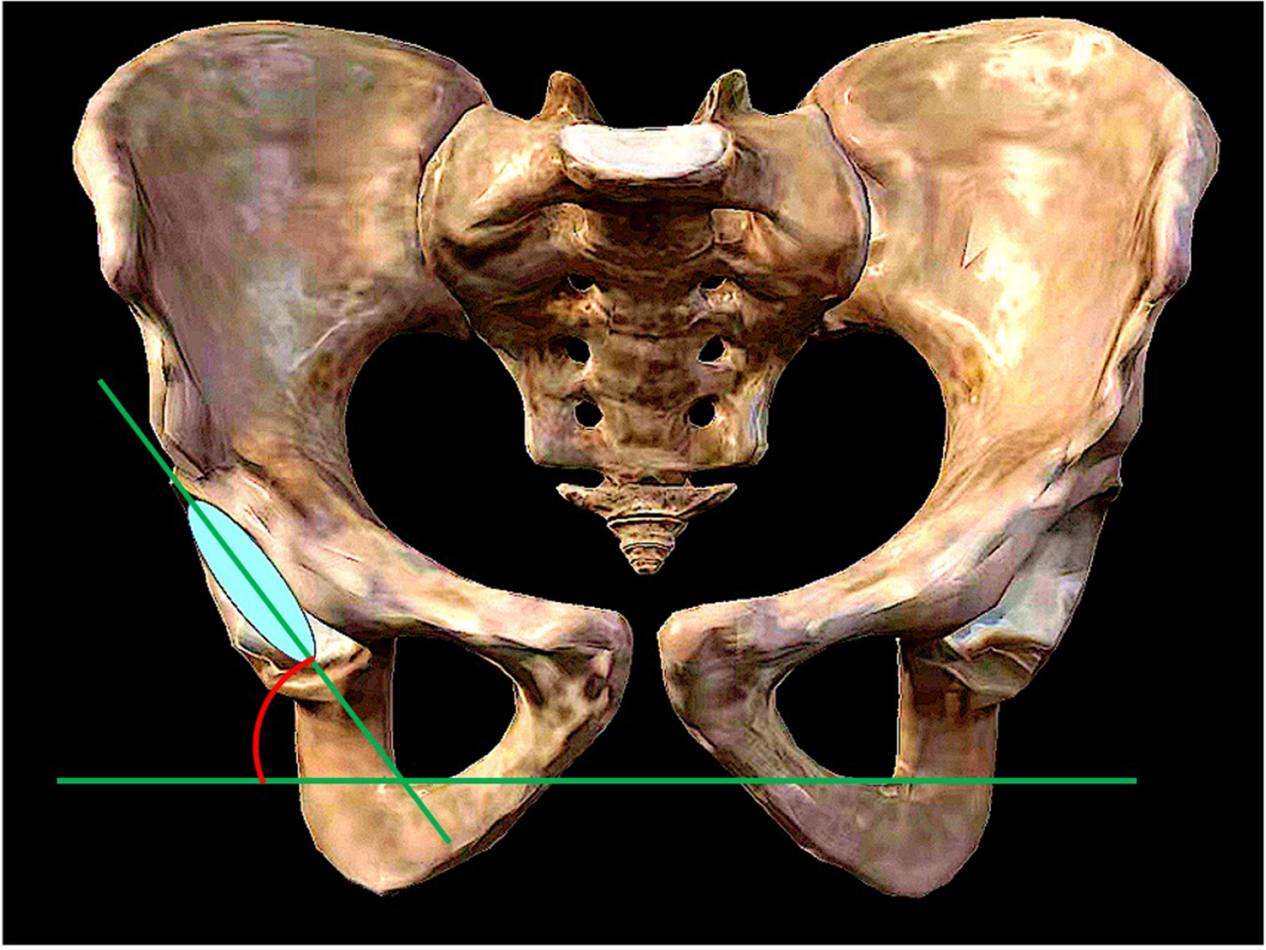
Acetabular shell inclination.

**Figure 2:**
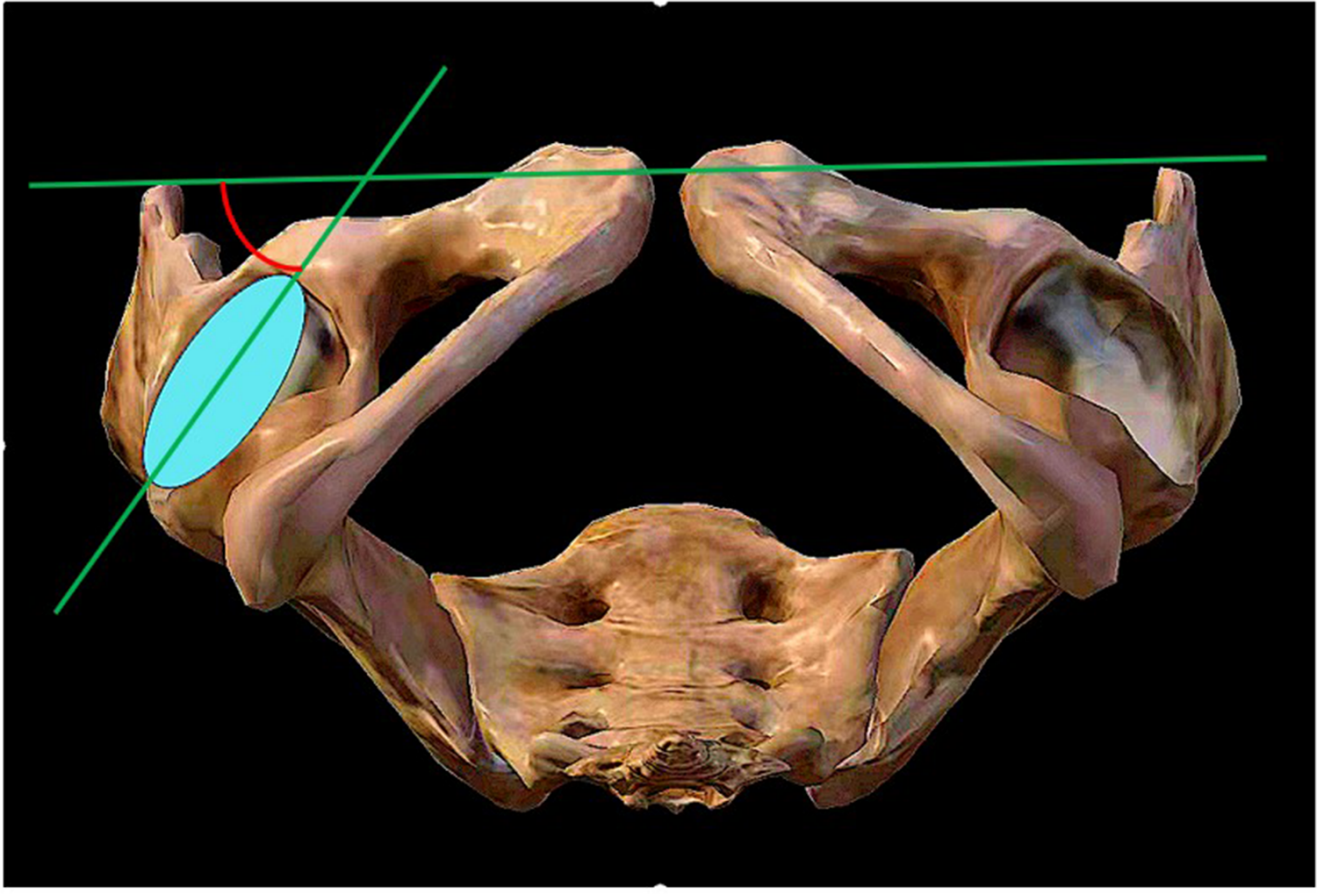
Acetabular shell anteversion.

**Figure 3:**
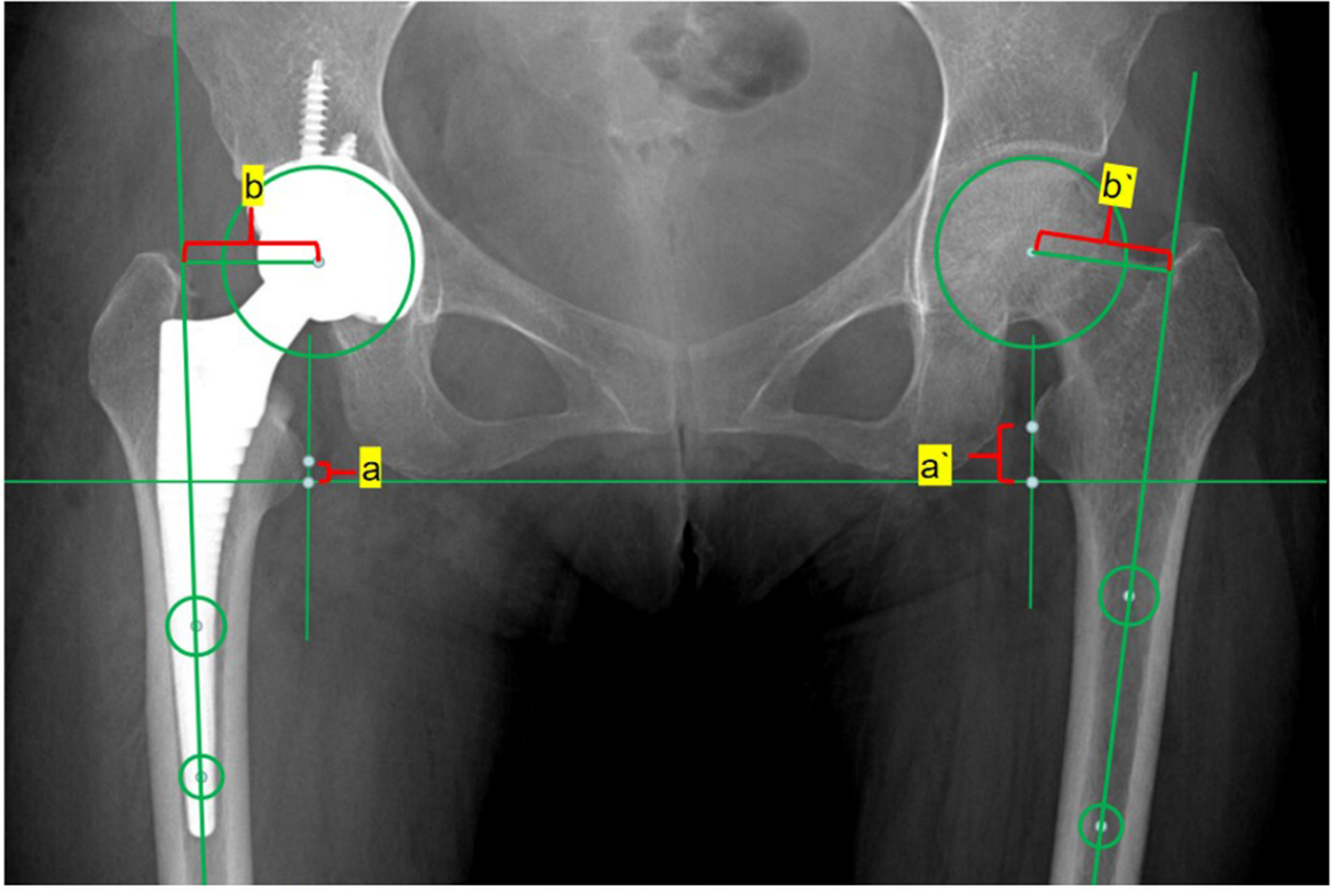
The LLD refers to the disparity between the distances from the bilateral lesser trochanter vertices to the bilateral ischial lines. LLD = |à-a|. The OD represents the disparity between the distance from the bilateral femoral head rotation centers to the extension line of the bilateral femoral shaft centers. OD = |b`-b|.

(1) Acetabular shell inclination: The angle formed between the diameter extension line of the acetabular shell and the frontal axis, as measured on the coronal plane of the bilateral hip joint using computed tomography (CT) (Figure 1).
(2) Acetabular shell anteversion: The angle formed between the diameter extension line of the acetabular shell and the sagittal axis, as measured on the transverse plane of the bilateral hip joint using CT (Figure 2).
(3) Femoral stem alignment: The angle formed between the femoral shaft axis, which serves as the longitudinal axis of the prosthesis, and the femoral longitudinal axis, as measured on the anterior-posterior and lateral radiographs of the bilateral hips. This determines whether the prosthesis is implanted in varus or valgus, flexion, or extension (9).
(4) Femoral stem anteversion: The angle formed between the centerline extension of the femoral stem neck and the coronal axis, as measured on the transverse plane of the bilateral hip joint using CT (9).
(5) Limb-length discrepancy: The difference in distance between the lower end of the ischial tuberosity and the most prominent inner point of the femoral lesser trochanter, as measured on the anteroposterior radiographs of the pelvis, between the affected and unaffected sides (9, 10) (Figure 3).
(6) Offset discrepancy: The difference in vertical distance between the center of rotation of the femoral head and the femoral long axis, as measured on the anterior-posterior view of the pelvis, between the affected and unaffected sides (9, 10) (Figure 3).

### Statistical methods

The data in this study were analyzed using IBM SPSS 26.0 software. Comparative analyses were conducted using independent samples *t*-tests for variables such as age, acetabular and femoral stem alignment. Chi-square tests were employed to analyze and compare variables like limb-length discrepancy (LLD), offset discrepancy, femoral head diameter, dislocation time, and other complications. One-way analysis of variance (ANOVA) was employed to compare the HHS scores between different groups. Continuous data were presented using mean ± standard deviation. A multifactorial logistic regression model was developed in order to identify independent risk factors for postoperative dislocation in patients. A *P*-value less than 0.05 was considered to be statistically significant.

The initial trial sample size calculation assumed a 20% subluxation rate for patients with LLD < 10 mm, 10% for patients with 10 mm < LLD < 20 mm, and 5% for LLD > 20 mm. A total of 256 patients needed to be included when the power of test and significance level were 0.80 and 0.05, respectively, as calculated by the PASS 2021 software. Therefore, the sample size of this study was adequate.

## Results

A total of 258 patients were included in the current study. Of all 258 patients, 35 patients were identified as having undergone postoperative prosthetic dislocation. Of the 35 patients with dislocation, isolated early dislocation, isolated late dislocation and recurrent dislocation were found in 23 patients, 5 patients and 7 patients respectively. The overall incidence of post hip replacement dislocation was 13.57%. The incidence and classification of post hip replacement dislocations are shown in Table 1.

**Table 1:** Baseline data of patients with neurological disorders undergoing total hip arthroplasty.

		Dislocation (*n* = 35)	Non-dislocation (*n* = 223)	*T*/*χ*^2^	*P*
Age		65.57 ± 6.099	63.71 ± 5.846	1.738	0.083
Sex	Male	17	116	0.114	0.704
	Female	18	107		
Indications	Femoral neck fracture	17	116	2.066	0.559
	Osteonecrosis	6	39		
	Osteoarthritis	8	50		
	Hip dysplasia	3	14		
	Other	0	0		
Type of neurological disorder	Ischemic stroke	13	79	2.920	0.404
	Hemorrhagic stroke	15	76		
	Poliomyelitis	5	32		
	Other	2	36		
Abductor strength	<Grade 2	15	110	0.893	0.64
	Grade 2	11	54		
	Grade 3	9	59		

### Demographic and general information

In the group with dislocations, there were 17 male and 18 female patients, while in the non-dislocation group, there were 116 male and 107 female patients (*χ*^2^ = 0.114, *P* = 0.704). There were no significant differences in age between the two groups (65.57 ± 6.099 years vs. 63.71 ± 5.846 years, *T* = 1.738, *P* = 0.083). No differences were observed between the two groups in terms of surgical indications (*χ*^2^ = 2.066, *P* = 0.559). There were no significant differences in the classification of neuromuscular diseases between the two groups (*χ*^2^ = 2.920, *P* = 0.404). Furthermore, no significant differences were found in terms of abduction muscle strength between the two groups (*χ*^2^ = 0.893, *P* = 0.64).

### Surgical characteristics

There were no significant differences in Acetabular shell inclination (*T* = 1.036, *P* = 0.301) and Acetabular shell anteversion (*T* = 1.036, *P* = 0.301) between the dislocation group and the non-dislocation group. Similarly, no significant differences were observed in Stem sagittal alignment (*T* = −1.000, *P* = 0.318) and Stem sagittal alignment (*T* = −1.227, *P* = 0.162) between the two groups. There were also no significant differences in Femoral stem anteversion (*T* = 0.664, *P* = 0.507). Overall, there were no significant differences in the composition of Limb-length discrepancy between the two groups (*χ*^2^ = 4.187, *P* = 0.123) Furthermore, no significant differences were found in the composition of Femoral head diameter between the two groups (*χ*^2^ = 0.635, *P* = 0.888). However, when comparing the dislocation group to the non-dislocation group, it was found that a higher proportion of patients in the dislocation group had a smaller Offset discrepancy (*χ*^2^ = 12.214, *P* = 0.002). The characteristics of the surgical procedure can be seen in Table 2.

**Table 2:** Surgical characteristics of patients with neurological disorders undergoing total hip arthroplasty.

		Dislocation (*n* = 35)	Non-dislocation (*n* = 223)	*T*/*Z*	*P*
Acetabular shell inclination (°)		37.629 ± 4.845	36.735 ± 4.726	1.036	0.301
Acetabular shell anteversion (°)		19.20 ± 5.235	18.525 ± 4.201	0.727	0.471
Femoral stem alignment (°)	Stem sagittal alignment	−0.286 ± 1.706	0.283 ± 1.713	−1.000	0.318
	Stem sagittal alignment	−0.256 ± 1.637	0.072 ± 1.360	−1.227	0.162
Femoral stem anteversion (°)		19.629 ± 2.734	19.2466 ± 3.224	0.664	0.507
Limb-length discrepancy (mm)	≤10	16 (45.7%)	78 (35%)	4.187	0.123
	>10, ≤20	14 (40%)	76 (34.1%)		
	>20	5 (14.3%)	69 (30.9%)		
Offset discrepancy (mm)	≤5	21 (60%)	72 (32.3%)	12.214	0.002
	>5, ≤10	10 (28.6%)	70 (31.4%)		
	>10	4 (11.4%)	81 (36.3%)		
Femoral head diameter	22 mm	2 (5.7%)	11 (4.9%)	0.635	0.888
	28 mm	7 (20%)	36 (16.1%)		
	32 mm	14 (40%)	104 (46.6%)		
	36 mm	12 (34.3%)	72 (32.3%)		

### Complications associated with different levels of limb-length discrepancy

The study findings revealed a close association between Limb-length discrepancy (LLD) and Offset discrepancy (OD) and the occurrence of postoperative complications. Based on the variations in Limb-length discrepancy and Offset discrepancy, the 258 patients were divided into three groups.According to the numerical differences in Limb-length discrepancy, all patients were categorized into three groups: LLD ≤ 10 mm, 10 mm < LLD ≤ 20 mm, and LLD > 20 mm. The incidence of early dislocation in these groups was 17%, 12.2%, and 4.1%, respectively (*χ*^2^ = 6.823, *P* = 0.033). A significant difference in dislocation rates was observed between patients with LLD ≤ 10 mm and LLD > 20 mm (OR = 0.206; 95% CI, 0.058–0.737). However, there were no significant differences among the three groups in terms of late dislocation incidence (*χ*^2^ = 0.322, *P* = 0.871). The occurrence rates of complications in patients with different levels of LLD were 3.2%, 11.1%, and 17.6%, respectively (*χ*^2^ = 9.606, *P* = 0.008). Again, a significant difference in overall complication rates was observed between patients with LLD ≤ 10 mm and LLD > 20 mm (OR = 6.464; 95% CI, 1.768–23.640). The Harris scores for the three groups were 81.436 ± 4.516, 81.614 ± 4.901, and 78.220 ± 4.323, respectively (*F* = 13.622, *P* < 0.001).The effect of limb lengthening are shown in Table 3.

**Table 3:** Effect of limb lengthening on the incidence of dislocation and the prognosis of patients.

			LLD ≤ 10 mm (*n* = 94)	10 mm < LLD ≤ 20 mm (*n* = 90)	LLD > 20 mm (*n* = 74)	*χ*^2^/*F*	*P*
Early dislocation (*n* = 30)	No		78 (83%)	79 (87.8%)	71 (95.9%)	6.823	0.033
	Yes		16 (17%)	11 (12.2%)	3 (4.1%)		
Late dislocation (*n* = 12)	No		90 (95.7%)	85 (94.4%)	71 (95.9%)	0.322	0.871
	Yes		4 (4.3%)	5 (5.6%)	3 (4.1%)		
Other complication	No	232	91 (96.8%)	80 (88.9%)	61 (82.4%)	9.606	0.008
	Overall	26	3 (3.2%)	10 (11.1%)	13 (17.6%)		
		Back pain	0 (0%)	1 (1.1%)	1 (1.4%)		
		Scoliosis	0 (0%)	2 (2.2%)	3 (4.1%)		
		Pelvic tilt	2 (2.1%)	6 (6.7%)	7 (9.5%)		
		Knee osteoarthritis	1 (1.1%)	1 (1.1%)	2 (2.7%)		
Harris score			81.436 ± 4.516	81.614 ± 4.901	78.220 ± 4.323	13.622	<0.001

### Complications associated with different levels of offset discrepancy

Based on the variations in Offset discrepancy, all patients were divided into three groups: OD ≤ 5 mm, 5 mm < OD ≤ 10 mm, and OD > 10 mm. The incidence of early dislocation in these groups was 17.2%, 12.5%, and 4.7%, respectively (*χ*^2^ = 6.837, *P* = 0.033). A significant difference in dislocation rates was observed between patients with OD ≤ 5 mm and OD > 10 mm (OR = 0.238; 95% CI, 0.076–0.742). For late dislocation incidence, the three groups had rates of 10.8%, 12.5%, and 0% (*χ*^2^ = 12.788, *P* = 0.002). There was no significant difference in complication occurrence rates among patients with different levels of OD (*χ*^2^ = 0.924, *P* = 0.63). The Harris scores for the three groups were 80.990 ± 5.232, 80.129 ± 4.697, and 80.543 ± 4.483, respectively (*F* = 0.685, *P* = 0.505). Refer to Table 4 for a comprehensive analysis of the effect of offset discrepancy.

**Table 4:** Effect of offset on the incidence of dislocation and the prognosis of patients.

			OD ≤ 5 mm (*n* = 93)	5 mm < OD ≤ 10 mm (*n* = 80)	OD > 10 mm (*n* = 85)	*χ*^2^/*F*	*P*
Early dislocation (*n* = 30)	No		77 (82.8%)	70 (87.5%)	81 (95.3%)	6.837	0.033
	Yes		16 (17.2%)	10 (12.5%)	4 (4.7%)		
Late dislocation (*n* = 12)	No		83 (89.2%)	78 (97.5%)	85 (100%)	12.788	0.002
	Yes		10 (10.8%)	2 (2.5%)	0 (0%)		
Other complication	No	232	86 (92.5%)	72 (90%)	75 (88.2%)	0.924	0.63
	Overall	26	7 (7.5%)	8 (10%)	10 (11.8%)		
		Back pain	1 (1.1%)	0 (0%)	1 (1.2%)		
		Scoliosis	1 (1.1%)	2 (2.5%)	4 (4.7%)		
		Pelvic tilt	5 (5.4%)	6 (7.5%)	3 (3.5%)		
		Knee osteoarthritis	2 (2.2%)	0 (0%)	2 (2.4%)		
Harris score			80.990 ± 5.232	80.129 ± 4.697	80.543 ± 4.483	0.685	0.505

### Independent risk factors

Four independent risk factors associated with postoperative dislocation in patients with muscle weakness have been ascertained through multifactorial logistic regression analysis. Of these factors, two are patient-related, namely age and abduction strength. The remaining two factors are intricately tied to the surgical procedure. The progression of age correlates with an augmented incidence of postoperative dislocation (OR = 1.021; 95% CI, 1.012–1.125), with older patients facing a heightened risk of dislocation. According to the hierarchical grading system elucidated by the Medical Research Council, patients with a muscular strength grade of 3 exhibit a diminished likelihood of postoperative dislocation when juxtaposed with individuals graded ≤2 (OR = 0.455; 95% CI, 0.238–0.987). Furthermore, patients with an Offset discrepancy (OD) measuring ≥10 mm demonstrate a reduced susceptibility to dislocation compared to those with an OD <5 mm (OR = 0.238; 95% CI, 0.076–0.742). Femoral head diameter additionally emerges as another risk factor intertwined with the surgical process. Patients with a diminutive femoral head diameter are more predisposed to postoperative prosthesis dislocation when contrasted with those employing a diameter of 36 mm (OR = 1.325; 95% CI, 1.125–3.277). The independent risk factors associated with postoperative dislocation in patients with muscle weakness summarized in Table 5.

**Table 5:** Independent risk factors associated with postoperative dislocation inpatients with insufficient muscle strength.

		OR	95% CI for OR	*P*
Age		1.021	1.012–1.125	0.002
Offset	OD ≤ 5 mm	Ref.		
	OD > 10 mm	0.238	0.076–0.742	<0.001
Abductor strength	≤Grade 2	Ref.		
	Grade 3	0.455	0.238–0.987	<0.001
Femoral head diameter	36 mm	Ref.		
	Other	1.325	1.125–3.277	0.015

## Discussion

Total hip arthroplasty (THA) has been proven to effectively reduce pain and improve quality of life in patients with neuromuscular diseases associated with hip joint disorders (11, 12). In a study by Yoon et al. (11), THA was performed on 10 patients with sequelae of cerebral palsy, and the results showed significant reduction in hip pain and improvement in function after the surgery. Compared to general hip joint disease patients, those with neuromuscular diseases often have muscle imbalance in the hip, making them more susceptible to prosthesis dislocation following THA (13). In this study, the overall incidence of prosthesis dislocation in patients with neuromuscular diseases after THA was 13.57%, higher than the dislocation rate in general patients undergoing THA. This is consistent with findings from other studies, which report dislocation rates ranging from 5% to 20% depending on the type of neuromuscular disease (14). In this study, all patients underwent a posterior-lateral approach. Although there are concerns that a posterior-lateral approach may increase the risk of prosthesis dislocation after THA, it actually provides a good surgical view for the surgeon to better understand the position of the acetabulum and femoral prosthesis. There is also evidence that repairing the posterior joint capsule can significantly reduce the risk of prosthesis dislocation (15). The study by Kwon et al. (16) showed that there seems to be no difference between different surgical techniques in most patients with neuromuscular diseases, and there is currently a diversity in the choice of prosthesis. The prostheses used in this study were all ceramic-on-ceramic materials, and Yoon et al. (12) reported good results when using ceramic-on-ceramic bearings.

Surgeons often face the challenge of increasing the length of the lower limbs in their pursuit of stable hip joints (17). However, this study indicates that it is not feasible to reduce the postoperative dislocation rate in patients with neuromuscular diseases by elongating the lower limbs. Although the elongation of limbs may decrease the early dislocation rate in patients, it may be attributed to the short-term increase in soft tissue tension caused by limb elongation, without improving the strength of the abductor muscles. Research has shown a negative correlation between the extent of leg lengthening and the strength of the abductor muscles (18, 19), which is one of the important factors affecting hip joint dislocation. Moreover, there is no consistent definition for the difference in limb length, ranging from 6 to 35 ml (20). Most studies consider differences less than 1 cm acceptable (21). However, as the difference in leg length increases, it can have biomechanical implications, leading to functional limitations in gait, posture, balance, and musculoskeletal disorders such as low back pain, scoliosis, and degenerative changes in the spine (22). This also explains why the Harris score of patients with limb length discrepancy (LLD) decreases with an increase in LLD one year postoperatively.

In this study, both early and late dislocations were found to be negatively correlated with the difference in eccentricity, which is consistent with previous research (23). We can posit that increasing the eccentricity difference effectively reduces the dislocation rate. Adequate soft tissue tension can increase the stability of the hip joint, with the abductor muscle group being the most critical. Increasing the offset can enlarge the transverse lever arm of the abductor muscles, thereby increasing the initial passive tension in the transverse direction, pulling the femoral prosthesis towards the center of the acetabulum. Meanwhile, there is no significant increase in the longitudinal lever arm, avoiding an increase in ineffective tension in the longitudinal direction. This also prevents a series of complications such as low back pain and pelvic tilt caused by unequal lengths of the lower limbs.

Through multifactorial logistic regression analysis, we have also discerned that the risk of THA dislocation escalates with advancing age and diminishing prosthetic head size. Advanced age has consistently been demonstrated as an independent risk factor for post-THA dislocation (24). Studies indicate that the utilization of a femoral head with a diameter greater than or equal to 32 mm can reduce the risk of dislocation by 35%–43% (25). Larger femoral head prostheses reside more deeply within the acetabular cup, necessitating a greater displacement for dislocation to occur.

This study still has limitations. Firstly, the collection of neuromuscular disease types in this study is not comprehensive, and there was no comparison of postoperative dislocation rates in different neuromuscular disease patients undergoing THA. The impact of certain neuromuscular diseases on postoperative dislocation rates in THA is currently controversial (26). Secondly, the use of ceramic-on-ceramic articulation in all the artificial biocompatible total hip joint prostheses used in this study did not include cases with other types of prostheses, thus failing to determine whether other types of hip joint prostheses have a relevant impact on the postoperative dislocation rates after hip arthroplasty. Thirdly, given the absence of established criteria for measuring abduction strength, this study employs a grading system for lower limb muscle strength (8) as a surrogate for assessing abduction strength.

Neuromuscular disease patients undergoing THA are at a higher risk of postoperative dislocation, which can occur repeatedly, ultimately leading to revisions (27, 28). Therefore, it is important to focus on reducing the occurrence of postoperative dislocation in these patients. By comparing the surgical data of neuromuscular disease patients with and without postoperative dislocation following THA, valuable references can be provided to surgeons, offering experimental evidence for selecting surgical approaches for such patients.

## Conclusions

Although increasing limb length can partially reduce the occurrence of early postoperative dislocation, it does not have an impact on the rate of late dislocation for patients with neurological diseases and muscle weakness. In addition, excessive limb lengthening is associated with other postoperative complications and worsening hip joint function. On the other hand, additional offset can decrease the likelihood of postoperative dislocation without increasing the incidence of other complications. Therefore, it is recommended to recommend femoral stems with a lower neck-shaft angle (higher offset) to patients with neurological diseases who are at a higher risk of postoperative dislocation. Pure limb lengthening should be avoided.

## Data Availability

The original contributions presented in the study are included in the article/Supplementary Material, further inquiries can be directed to the corresponding author.
